# “What Goes Up Must Come Down”: Laparoscopic Retrieval of a Migrated Intrathoracic Kidney and Repair of Recurrent Symptomatic Diaphragmatic Hernia

**DOI:** 10.1089/cren.2018.0001

**Published:** 2018-08-01

**Authors:** Nisha Pindoria, Jonathan Makanjuola, Abrie Botha, Rajesh Nair, Ramesh Thurairaja

**Affiliations:** ^1^ Department of Urology, Guy's and St Thomas' NHS Foundation Trust, Guy's Hospital, London, United Kingdom.; ^2^ Department of Upper GI Surgery, Guy's and St Thomas' NHS Foundation Trust, Guy's Hospital, London, United Kingdom.

**Keywords:** ectopic intrathoracic kidney, Bochdalek-type congenital diaphragmatic hernia, primigravida

## Abstract

***Background:*** Congenital diaphragmatic hernia is a rare condition describing a developmental defect of the diaphragm. It is managed surgically in the neonatal period by reduction of the herniated viscera followed by repair of the defect. We present a laparoscopic repair of a Bochdalek diaphragmatic hernia recurrence with retrieval and nephropexy of a migrated kidney with reduced function from its ectopic thoracic position. The complexities of managing this rare occurrence and lessons from this surgical challenge are discussed.

***Case Presentation:*** A 21-year-old primigravida presented with a 3-day history of right upper quadrant pain and increasing dyspnea. Of note, she had undergone a congenital right-sided diaphragmatic hernia repair as an infant. An MRI revealed a recurrent diaphragmatic defect with ectopic migration of the right kidney and bowel into an intrathoracic position. Due to worsening dyspnea, she underwent prompt laparoscopic repair of her recurrent diaphragmatic hernia. Subsequently, she underwent a planned cesarean section to control her intra-abdominal pressures and reduce the risk of hernia repair failure.

***Conclusion:*** Raised intra-abdominal pressures during pregnancy in patients with prior congenital hernia repair can result in recurrence and migration of peritoneal and retroperitoneal contents into the chest. In cases of renal unit migration, the primary concern must be to restore the anatomical position of a functioning kidney. Multidisciplinary specialist involvement in a tertiary referral base is crucial to an effective outcome.

## Introduction and Background

Congenital diaphragmatic hernia (CDH) is a rare condition describing a developmental defect of the diaphragm affecting between 1:2000 and 1:4000 live births annually. It is characterized by pulmonary hypoplasia and hypertension associated with decreased pulmonary vasculature and alterations in surfactant system resulting in respiratory distress. Surgical correction is performed in the neonatal period by reduction of the herniated viscera followed by repair of the diaphragmatic defect.^1^ Recurrence, although rare, can result from intraoperative or postoperative factors. We describe a case of late recurrence and migration of the kidney from an intra-abdominal to thoracic location in a patient with raised intra-abdominal pressures secondary to pregnancy.

## Presentation of Case

A 21-year-old Caucasian in her second trimester of pregnancy presented with a 3-day history of intermittent sharp right upper quadrant (RUQ) pain and increasing dyspnea. Of note, she had previously undergone a right-sided CDH repair at 12 days after birth. On clinical examination, she had equal air entry of her chest, and an abdominal examination revealed RUQ tenderness with no features of peritonitis. Blood parameters revealed no evidence of anemia, renal impairment, or infection. A chest X-ray performed due to worsening dyspnea and RUQ pain revealed herniation of bowel within the thoracic cavity on the right side. Subsequently, an MRI of the thorax and abdomen confirmed a right diaphragmatic defect with ectopic migration of the right kidney to the posterior mediastinum. This was in addition to migration of the colon and small bowel lateral to the liver within the thorax. A recurrence of the previous right Bochdalek CDH secondary to raised intra-abdominal pressures from pregnancy resulted. The patient remained symptomatic with shortness of breath and difficulty in maintaining oral intake. Consequently, a multidisciplinary team discussion, involving general surgeons, cardiothoracic surgeons, urologists, and obstetricians, deemed urgent diaphragmatic repair was necessary to prevent potentially life-threatening complications of visceral herniation and improve symptoms. Furthermore, anatomical relocation of the kidney was fundamental to preserve renal function and obviate mother and fetal morbidity associated with progressive renal impairment.

**FIG. 1.**
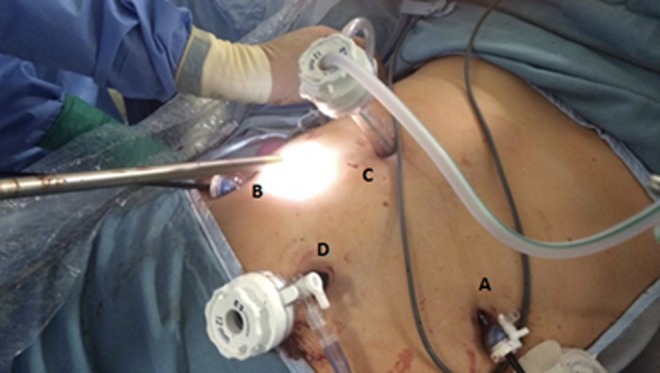
Intraoperative image demonstrating port position. **(A)** Camera port positioned superior and medial to the left anterior superior iliac spine, **(B)** working port positioned superior and lateral to the right anterior superior iliac spine, **(C)** working port positioned at the right costal margin, and **(D)** working port positioned superior and lateral to the umbilicus.

**FIG. 2.**
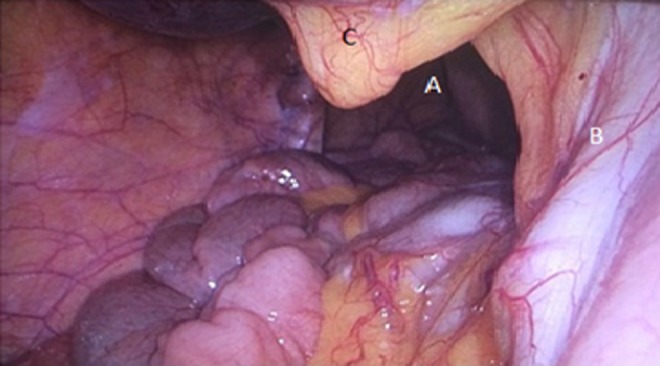
Intra-abdominal laparoscopic view demonstrating **(A)** diaphragmatic defect with small bowel herniation, **(B)** right renal ureter and vessels adherent over the right crus of the diaphragm, and **(C)** lower pole of right perinephric tissue.

**FIG. 3.**
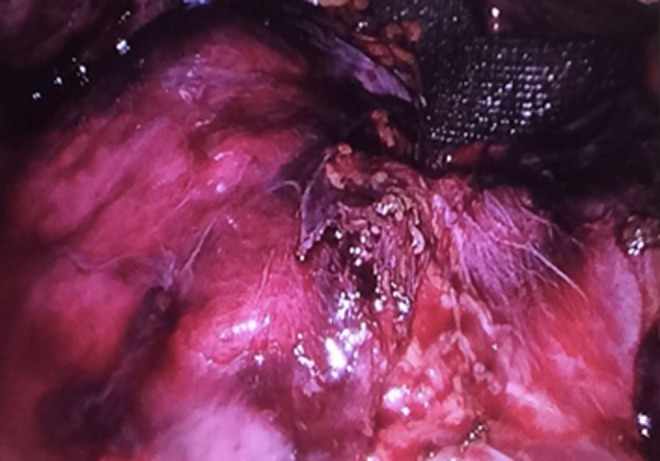
Intra-abdominal laparoscopic views of prosthetic mesh repair placed over the diaphragmatic defect.

**FIG. 4.**
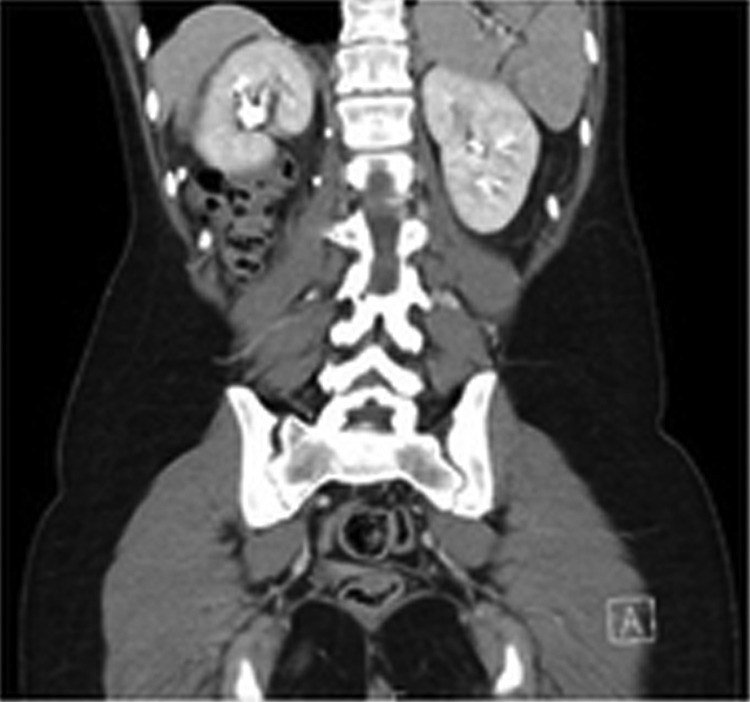
Postpartum CT demonstrating repair of the Bochdalek CDH and the appropriately positioned right kidney within the abdomen posterior to the right lobe of the liver. CDH, congenital diaphragmatic hernia.

Before surgery, advice from cardiothoracic surgeons was sought to identify the most suitable laparoscopic approach (intra-abdominal *vs* thoracoscopic). Due to difficulty in identifying renal vessels originating from the intra-abdominal cavity, the general consensus was to obtain control of the ureter and renal vessels as a priority, preceding reduction of herniated contents and closure of the diaphragmatic defect with mesh. As a result, the first step involved delineating the course of the ureter with cystoscopy and retrograde stent insertion using ultrasound guidance. Once the proximal aspect of the stent was confirmed within the renal pelvis, the patient was positioned in the left lateral position and four ports were inserted, including a camera port and three working ports. The camera port was positioned superior and medial to the left anterior superior iliac spine. The three working ports were positioned superior and lateral to the right anterior superior iliac spine, at the right costal margin, and superior and lateral to the umbilicus (Fig. 1). An insufflation pressure of 10 mmHg was used. An obstetric anesthetist was involved in monitoring the patient, while obstetricians and cardiothoracic surgeons were available on standby. Following ureteral stent insertion, it was possible to identify the renal artery and vein running alongside it. The vessels were followed up until the renal hilum was reached, which was then mobilized (Fig. 2). Nylon tape was used to isolate the vessels and ureter to protect them from iatrogenic injury. The next step involved mobilization of the bowel using Maryland graspers. In conjunction, the anesthetist positioned the patient head up and performed a Valsalva maneuver, increasing intrathoracic pressure to facilitate in reduction of bowel loops into the intra-abdominal cavity. A prosthetic mesh patch was utilized for closure of the diaphragmatic defect (Fig. 3). Following this, nephropexy was performed using absorbable 2/0 PDS. The superior pole of the kidney was anchored to the superior lateral abdominal wall, the posterior aspect to the origin of the diaphragmatic defect, and the medial aspect to the prosthetic mesh. On closure, an abdominal drain was inserted. An inadvertent right pneumothorax was created during mobilization of her bowels and kidney from the pleura. This was effectively managed with a chest drain. Postoperative recovery was uneventful with both her abdominal and chest drain removed 2 days postoperatively and she was subsequently discharged after 1 week. An elective flexible cystoscopy to remove the ureteral stent was performed 2 weeks from the date of procedure. A postpartum CT demonstrated the repaired Bochdalek CDH and the appropriately positioned right kidney within the abdomen posterior to the right lobe of the liver (Fig. 4). Her pregnancy continued to term, where she underwent an elective cesarean section to reduce the risk of hernia repair failure. A postpartum MAG3 renogram revealed a 55% and 45% split function of the right and left kidney, respectively.

## Discussion and Literature Review

Of the three types of CDH, Bochdalek is the most common, contributing to 85% of cases. The Bochdalek-type hernia results from the developmental failure of the posterolateral foramina to fuse. Interestingly, the vast majority of Bochdalek hernias affect the left side (80%–90%). Right-sided defects are rare with less than 50 reported cases in the literature. Here the right pleuroperitoneal canal closes prematurely and the liver buttresses the right diaphragm. CDH resulting in intrathoracic renal ectopia is an extremely rare developmental anomaly and the rarest form of all ectopic kidneys, associated with an incidence of 1 in 10,000 cases.^2^ To our knowledge, this is the first reported case of symptomatic CDH with renal ectopic migration in pregnancy, requiring urgent intervention.

A previous case report of symptomatic left-sided CDH in pregnancy involving intrathoracic bowel herniation has been reported. An open left thoracoabdominal approach was used and evidence of strangulation and gangrene of the transverse colon found. A segmental resection of large intestine was performed and the diaphragmatic defect repaired with interrupted sutures. An uneventful elective cesarean section was then performed to prevent raised abdominal pressures associated with vaginal delivery.^3^

In this particular case, raised intra-abdominal pressures associated with pregnancy resulted in subsequent migration of peritoneal and retroperitoneal contents and significant symptoms for our patient. Other factors attributing to hernia recurrence include method of hernia defect closure (suture *vs* mesh, absorbable sutures *vs* nonabsorbable sutures), increased tension during primary closure, improper fixation of prosthetic mesh material, and increase in abdominal pressure secondary to ileus or poor chest toileting.^1^

Simple intrathoracic renal ectopia is usually asymptomatic and diagnosed incidentally. Therefore, in asymptomatic pregnant patients, CDH can be managed at the time of birth to avoid complications of surgery in pregnancy. In such cases, an elective cesarean section with concurrent surgical repair should be performed to prevent increase in abdominal pressure and worsening of the diaphragmatic hernia associated with bearing down in vaginal delivery. However, when symptoms arise, prompt surgical intervention is necessary to prevent severe and life-threatening complications.

Significant complications that may arise include strangulation of herniated organs and difficulty in ventilation with compression atelectasis of the lungs secondary to pressure-related effects of migrated organs.^3^ In the event of renal unit migration, restoration of anatomical position is key to kidney function preservation and prevention of long-term renal impairment. In cases involving previous abdominal surgery, an open surgical approach may be regarded as optimal to minimize tedious and difficult dissections arising from adhesions. However, the laparoscopic technique is being increasingly adopted to treat hernia recurrence due to its inherent associations with reduced postoperative pain. Furthermore, trials have demonstrated no apparent increase in risk of recurrence rates with laparoscopic *vs* open methods. The diaphragmatic defect can be repaired with interrupted sutures if it is small or prosthetic mesh patch in large defects. However, with recurrent CDH, a mesh repair is favored regardless of size to minimize the probability of rerecurrence.^2^ There are concerns that during pregnancy the growing fetus can increase intra-abdominal pressure disrupting the mesh repair. Indeed, there has been a case report of spontaneous rupture of hernial repair during pregnancy.^4^ To minimize the risk of hernia repair failure, the patient underwent an elective cesarean section at 39 weeks of gestation, minimizing the risk of increased abdominal pressures associated with vaginal delivery. This case illustrates a number of salient points when managing an incredibly rare case complicated by pregnancy. Access to multidisciplinary specialist involvement with consideration of timings of surgery (during gestation *vs* postpartum), surgical technique (open *vs* laparoscopic), and postoperative follow-up are crucial to optimize outcomes. Fundamentally, the potential risk of harm or fetal miscarriage associated with operative intervention, as well as the effects of a growing fetus on postoperative outcomes, requires careful counseling with parents.

## Conclusion

Raised intra-abdominal pressures during pregnancy in patients with prior congenital hernia repair can result in recurrence and migration of peritoneal and retroperitoneal contents into the chest. In cases of renal unit migration, primary concern must be to restore the anatomical position of a functioning kidney. Multidisciplinary specialist involvement in a tertiary referral base is crucial to an effective outcome.

## Abbreviations Used

CDH: congenital diaphragmatic hernia
CT: computed tomography
MRI: magnetic resonance imaging
PDS: polydioxanone
RUQ: right upper quadrant
